# Sales of antibiotics without a prescription in pharmacies, 2017 and 2021, China

**DOI:** 10.2471/BLT.22.289435

**Published:** 2023-03-21

**Authors:** Jie Chen, Ying Xie, Yiting Sun, Ruichen Zang, Yuhao Sun, Lintao Dan, Xuanding Wang, Therese Hesketh

**Affiliations:** a Centre for Global Health, Zhejiang University School of Medicine, 866 Yuhangtang Road, Hangzhou 310058, China.; b School of Health Policy and Management, Chinese Academy of Medical Sciences and Peking Union Medical College, Beijing, China.; c Department of Antimicrobial Stewardship, The Second Affiliated Hospital of Zhejiang University School of Medicine, Hangzhou, China.

## Abstract

**Objective:**

To measure sales of antibiotics without a prescription in pharmacies in China in 2017 and 2021, before and during the coronavirus disease 2019 (COVID-19) pandemic, and determine the factors associated with such sales.

**Methods:**

We conducted cross-sectional surveys using the simulated patient method in retail pharmacies in 13 provinces in eastern, central and western China in 2017 and 2021. At the pharmacies, the simulated patients (trained medical students) reported that they had mild respiratory tract symptoms and asked for treatment, using a three-stage process: (i) request some treatment; (ii) request antibiotics; (iii) request specific antibiotics. We used multivariable logistic regression analysis to determine factors associated with sale of antibiotics without a prescription.

**Findings:**

Of the pharmacies visited in 2017, 83.6% (925/1106) sold antibiotics without a prescription; this figure was 78.3% (853/1090) in 2021 (*P*-value: 0.002). After excluding pharmacies prohibited from selling antibiotics because of COVID-19, this difference was not significant (83.6% versus 80.9%; 853/1054; *P*-value: 0.11). Factors significantly associated with selling antibiotics without a prescription in both 2017 and 2019 were: location in central and western China compared with eastern China; being in a township or village compared with in a city; and presence of a counter where antibiotics were dispensed.

**Conclusion:**

Although laws became stricter between 2017 and 2021, antibiotic sales without a prescription were still common in pharmacies across China. Existing regulations need to be more strictly enforced, and pharmacy staff and the public should be made more aware of the risks of antibiotic misuse and dangers of antimicrobial resistance.

## Résumé

**Objectif:**

Mesurer les ventes d'antibiotiques sans ordonnance dans les pharmacies de Chine en 2017 et 2021, avant et pendant la pandémie de maladie à coronavirus 2019 (COVID-19), et déterminer les facteurs associés à ces ventes.

**Méthodes:**

Nous avons mené des enquêtes transversales à l'aide de la technique des patients simulés dans des pharmacies de détail situées dans 13 provinces de Chine orientale, centrale et occidentale en 2017 et 2021. Dans ces pharmacies, les patients simulés (des étudiants en médecine formés à cet effet) ont évoqué des symptômes légers au niveau des voies respiratoires et sollicité un traitement selon un processus en trois étapes: (i) demande de traitement; (ii) demande d'antibiotiques; (ii) demande d'antibiotiques spécifiques. Nous avons ensuite utilisé une analyse multivariée de régression logistique afin d'identifier les facteurs liés à la vente d'antibiotiques sans ordonnance.

**Résultats:**

Sur l'ensemble des pharmacies examinées en 2017, 83,6% (925/1106) ont vendu des antibiotiques sans ordonnance, contre 78,3% (853/1090) en 2021 (valeur-p: 0,002). Après avoir exclu les pharmacies n'ayant pas le droit d'en vendre à cause de la COVID-19, cette différence s'est révélée négligeable (83,6% contre 80,9%; 853/1054; valeur-p: 0,11). Les principaux facteurs associés à la vente d'antibiotiques sans ordonnance, tant en 2017 qu'en 2019, étaient: le fait d'être en Chine centrale et occidentale plutôt qu'en Chine orientale; le fait d'être une commune ou un village plutôt qu'une ville; et enfin, le fait qu'il y ait ou non un comptoir où se procurer des antibiotiques.

**Conclusion:**

Bien que les lois soient devenues plus strictes entre 2017 et 2021, la vente d'antibiotiques sans ordonnance est restée monnaie courante partout en Chine. La réglementation en vigueur doit être appliquée plus sévèrement; en outre, le personnel des pharmacies et le grand public doivent être mieux sensibilisés aux risques liés à un usage inapproprié des antibiotiques et aux dangers que représente la résistance aux antimicrobiens.

## Resumen

**Objetivo:**

Medir las ventas de antibióticos sin receta en farmacias de China en 2017 y 2021, antes y durante la pandemia de la enfermedad por coronavirus de 2019 (COVID-19), y determinar los factores asociados a estas ventas.

**Métodos:**

Se realizaron encuestas transversales utilizando el método del paciente simulado en farmacias minoristas de 13 provincias de China oriental, central y occidental en 2017 y 2021. En las farmacias, los pacientes simulados (estudiantes de medicina entrenados) informaron que tenían síntomas leves del tracto respiratorio y solicitaron tratamiento, mediante un proceso de tres etapas: (i) solicitar algún tratamiento; (ii) solicitar antibióticos; (ii) solicitar antibióticos específicos. Se utilizó un análisis de regresión logística multivariable para determinar los factores asociados a la venta de antibióticos sin receta.

**Resultados:**

De las farmacias visitadas en 2017, el 83,6% (925/1106) vendían antibióticos sin receta; esta cifra fue del 78,3% (853/1090) en 2021 (valor de *p*: 0,002). Una vez excluidas las farmacias que tenían prohibida la venta de antibióticos debido a la COVID-19, esta diferencia no fue significativa (83,6% frente a 80,9%; 853/1054; valor de *p*: 0,11). Los factores que se asociaron significativamente con la venta de antibióticos sin receta tanto en 2017 como en 2019 fueron: la ubicación en el centro y occidente de China en comparación con el oriente del país; estar en un municipio o pueblo en comparación con estar en una ciudad; y la presencia de un mostrador donde se dispensaban antibióticos.

**Conclusión:**

Aunque las leyes se hicieron más estrictas entre 2017 y 2021, la venta de antibióticos sin receta seguía siendo habitual en las farmacias de toda China. Es necesario aplicar más estrictamente la normativa vigente, y concienciar al personal de las farmacias y al público en general sobre los riesgos del uso indebido de antibióticos y los peligros de la resistencia a los antimicrobianos.

## ملخص

الغرض قياس مبيعات المضادات الحيوية من دون وصفة طبية في الصيدليات في الصين في عامي 2017 و2021، قبل وأثناء جائحة مرض فيروس كورونا 2019 (كوفيد 19)، وتحديد العوامل المرتبطة بمثل هذه المبيعات.

الطريقة قمنا بإجراء مسوحات مقطعية باستخدام طريقة محاكاة المريض، في صيدليات البيع بالتجزئة في 13 مقاطعة في شرق ووسط وغرب الصين، في عامي 2017 و2021. أوضح مرضى المحاكاة (طلاب الطب المدربون) في الصيدليات أنهم يعانون من أعراض متوسطة في الجهاز التنفسي، وطلبوا الحصول على علاج، باستخدام عملية من ثلاث مراحل: (1) طلب بعض العلاج؛ و(2) طلب المضادات الحيوية؛ و(3) طلب مضادات حيوية معينة. استخدمنا تحليل التحوّف اللوجستي متعدد المتغيرات لتحديد العوامل المرتبطة ببيع المضادات الحيوية دون وصفة طبية.

النتائج من بين الصيدليات التي تمت زيارتها في عام 2017، باعت %83.6 منها (925/1106) مضادات حيوية دون وصفة طبية؛ وكان هذا الرقم %78.3 (853/1090) في عام 2021 (نسبة الاحتمال: 0.002). بعد استبعاد الصيدليات المحظورة من بيع المضادات الحيوية بسبب كوفيد 19، لم يكن هذا الاختلاف ملموسًا (%83.6 مقابل %80.9؛ 853/1054؛ نسبة الاحتمال: 0.11). كانت العوامل المرتبطة بشكل ملموس ببيع المضادات الحيوية دون وصفة طبية في عامي 2017 و2019 هي: الموقع في وسط وغرب الصين مقارنة بشرق الصين؛ وأن تكون في بلدة أو قرية مقارنة بالمدينة؛ ووجود مكان يتم فيه صرف المضادات الحيوية.

الاستنتاج على الرغم من أن القوانين أصبحت أكثر صرامة بين عامي 2017 و2021، إلا أن بيع المضادات الحيوية دون وصفة طبية كان لا يزال شائعًا في الصيدليات في جميع أنحاء الصين. تحتاج اللوائح الحالية للتطبيق بشكل أكثر صرامة، ويجب أن يكون فريق عمل الصيدليات والجمهور أكثر وعيًا بمخاطر إساءة استخدام المضادات الحيوية، ومخاطر مقاومة مضادات الميكروبات.

## 摘要

**目的:**

旨在衡量 2017 年和 2021 年，即 2019 新型冠状病毒病肺炎 (COVID-19) 疫情之前和疫情期间中国药店无处方销售抗生素的情况，并确定与此类销售相关的因素。

**方法:**

2017 年和 2021 年，我们在中国东部、中部和西部 13 个省份的零售药店使用假装患者身份的方法进行了横断面调查。在药店，假装患者身份的人员（经过培训的医学生）报告称他们有轻微的呼吸道症状，并通过三个阶段的流程要求药店提供治疗药物：(i) 要求某些治疗药物；(ii) 要求使用抗生素；(ii) 要求使用特定的抗生素。我们使用多变量逻辑回归分析来确定与无处方销售抗生素相关的因素。

**结果:**

在 2017 年走访的药店中，83.6% (925/1106) 的药店在没有处方的情况下销售抗生素；这一数字在 2021 年变为 78.3% (853/1090)（*P* 值：0.002）。在排除因新型冠状病毒肺炎疫情而禁止销售抗生素的药店后，这种差异并不显著（即 83.6% 与 80.9%；853/1054；*P* 值：0.11）。在 2017 年和 2019 年，与无处方销售抗生素这一情况显著相关的因素是：与东部相比，多发生在中国中部和西部；与在城市相比，多发生在乡镇或村庄；以及多发生在设有处方抗生素药物柜台的药店。

**结论:**

尽管在 2017 年至 2021 年期间，相关法律越来越严格，但在中国各地的药店中，无处方销售抗生素的情况仍然很普遍。需要更加严格得执行现有法规，并且药店工作人员和公众应进一步了解抗生素滥用的风险和抗生素耐药性的危险。

## Резюме

**Цель:**

Измерить продажи антибиотиков без рецепта в аптеках Китая в 2017 и 2021 годах (до и во время пандемии коронавирусной болезни 2019 г. (COVID-19)) и определить сопутствующие факторы таких продаж.

**Методы:**

Были проведены перекрестные опросы с использованием метода имитации пациента в розничных аптеках в 13 провинциях Восточного, Центрального и Западного Китая в 2017 и 2021 гг. В аптеках имитируемые пациенты (подготовленные студенты-медики) сообщали о наличии у них легких симптомов со стороны респираторного тракта и обращались за лечением посредством трехэтапного процесса: (i) запрос определенного лечения; (ii) запрос антибиотиков; (iii) запрос конкретных антибиотиков. Для определения связанных с продажей антибиотиков без рецепта факторов использовался многомерный логистический регрессионный анализ.

**Результаты:**

Из посещенных в 2017 году аптек 83,6% (925/1106) продавали антибиотики без рецепта; в 2021 году этот показатель составил 78,3% (853/1090) (P-значение: 0,002). После исключения аптек, которым запрещалось продавать антибиотики из-за COVID-19, эта разница перестала быть существенной (83,6% против 80,9%; 853/1054; P-значение: 0,11). К числу факторов, в значительной степени связанных с продажей антибиотиков без рецепта в 2017 и 2019 гг., относятся: расположение в регионах Центрального и Западного Китая по сравнению с Восточным Китаем; проживание в поселке или деревне по сравнению с городом; наличие прилавка, где отпускаются антибиотики.

**Вывод:**

Несмотря на ужесточение законов в период с 2017 по 2021 год, продажа антибиотиков без рецепта по-прежнему часто встречается в аптеках по всему Китаю. Существующие правила должны соблюдаться более строго, а персонал аптек и население должны быть более информированы о рисках неправильного использования антибиотиков и опасностях, связанных с устойчивостью к противомикробным препаратам.

## Introduction

Antimicrobial resistance is one of the most serious threats to the health of the world’s population.^1^^,^^2^ In 2019, antimicrobial resistance was associated with 4.95 million deaths globally.^3^ Estimates suggest that antimicrobial resistance could lead to about 10 million deaths a year by 2050 unless urgent measures are taken.^2^ Misuse of antibiotics in agriculture and health care is the primary driver of antimicrobial resistance.^4^^,^^5^ In health care, antimicrobial stewardship programmes targeting doctors’ prescribing have been introduced in many countries with some success.^6^ For instance, in China, a national campaign on the rational use of antibiotics was conducted in hospitals, which resulted in a 10% decrease in prescriptions with antibiotics in both inpatient and outpatient settings.^7^ However, globally, nearly half of antibiotics were purchased without prescription from retail pharmacies.^8^^–^^11^

China is one of the world’s largest consumers and producers of antibiotics, and one of the drivers of antimicrobial resistance globally.^12^ In a national survey in 2020, the resistance rates of some bacteria were high nationwide. For example, erythromycin resistance was reported in 96.0% (73 355/76 412) of strains of *Streptococcus pneumoniae*.^13^ Apart from some topical antibiotics, such as chlortetracycline and erythromycin which are over-the-counter medicines, the law states that most antibiotics need to be purchased on prescription in China. Recent studies have shown that most retail pharmacies in China sold antibiotics without a prescription to simulated patients describing mild symptoms.^14^^–^^18^ The main types of antibiotics offered were penicillins (36.0%; 333/925), cephalosporins (29.6%; 274/925) and macrolides (27.0%; 250/925).^15^

Concerns about the growing problem of sales of antibiotics without a prescription have led to action by the Chinese government (see online repository).^19^ In 2003, policies on antimicrobial resistance were issued, including a ban on the sale of antibiotics without a prescription.^20^ In 2011, the health ministry established a task force on antibiotic stewardship in hospitals, which resulted in small reductions in antibiotic prescribing, especially in tertiary hospital settings.^21^ In 2012, the twelfth five-year plan on drug safety proposed that a licensed pharmacist guide the rational use of medicines and monitor drug use, including addressing the inappropriate use of antibiotics.^22^ In 2016, at the G20 summit, China affirmed the importance of tackling antimicrobial resistance. The Chinese government issued the National Action Plan with the goal of achieving all sales of antibiotics with a prescription in pharmacies by 2020.^23^ From 2011 to 2017, a decrease in sales of antibiotics without a prescription was observed in China, especially in Shanxi province where multiple initiatives had been taken.^14^ In 2020, further controls were announced, including that retail pharmacies selling antibiotics without prescriptions would be penalized more severely,^24^ with a steep increase in fines from about 1000 renminbi (¥), equivalent to 140 United States dollars (US$),^25^ to ¥ 30 000 (US$ 4215).^24^ This measure mirrors policies in other countries, where fines for selling antibiotics without prescriptions have been introduced, and where effectiveness depends on the degree of enforcement and the size of the fine.^26^^–^^29^

Efforts to tackle antimicrobial resistance have been complicated by the coronavirus disease 2019 (COVID-19) pandemic. During the first wave of the pandemic in 2020, estimates suggest that, globally, about half of hospitalized patients with COVID-19 died of bacterial or fungal infections.^27^ Antibiotics became widely used as part of the routine treatment for COVID-19,^28^ although limited evidence was available of their effect on health outcomes.^29^ Reports from 2022, much later in the course of the pandemic, indicated that only 2.5% (25/989) to 5.1% (21/408) of hospitalized patients with COVID-19 were co-infected with bacteria.^30^

In some areas of China, during local COVID-19 outbreaks, drug sales for respiratory symptoms were temporarily banned (usually for 1–2 months) in retail pharmacies. These medicines included cough suppressants, in addition to antibiotics.^31^^,^^32^ The reason for this ban was because self-medication was thought to delay the diagnosis of COVID-19, thus increasing the risk of its spread in the population.^20^

Several studies exploring the sale of antibiotics without a prescription in China were conducted before the outbreak of COVID-19.^14^^,^^16^^–^^18^ In this study, we aimed to: (i) compare the prevalence of antibiotic sales without a prescription in 2017 and 2021, before and after the new stricter regulations introduced during COVID-19 in China; and (ii) explore the characteristics of the pharmacies where antibiotics were sold without a prescription, as well as the quality of service during antibiotic sales.

## Methods

We used the same method for both the 2017 and 2021 surveys, that is, a cross-sectional study using the simulated patient method. We conducted the surveys in the same 13 provinces in the three geographic regions of mainland China (see online repository).^19^

We used a multistage sampling process in both surveys. We used purposive sampling to select four to five provinces each in the eastern, central and western regions of China to broadly represent socioeconomic status. In each province, we selected the capital city, one small city and one county. The small cities and counties were different in the two surveys. We selected at least 80 pharmacies per province and all pharmacies were within 10 km of the simulated patient’s home.

### Categories of pharmacies

To ensure a broad representation of types of pharmacy, we purposively selected pharmacies within three categories. We used Google or Baidu maps for location information.^15^

#### Administrative level

We categorized pharmacies according to administrative level: city, county, or township and village. Cities were urban areas, townships and villages were rural areas, and counties were rural regions with urban characteristics. A previous study assumed that it may be harder to obtain antibiotics without a prescription in urban areas where policies on sales of antibiotics are more likely to be enforced than in than rural areas.^15^

#### Distance from a hospital

We categorized pharmacies as being within 2 km of a hospital or more than 2 km of a hospital. Previous research showed that pharmacies near a hospital may be less likely to offer antibiotics without a prescription because prescriptions are more readily available at the hospital.^15^

#### Type of pharmacy

We categorized pharmacies as independent or part of a chain of pharmacies. We assumed that independent pharmacies were less regulated and so may be more likely to sell antibiotics without a prescription.^15^

### Procedures

We used the simulated patient method in both surveys. We recruited 100 undergraduate medical students and trained them to act as simulated patients (49 students in 2017 and 51 students in 2021). The students were from Zhejiang University, Xiamen University, Xiangya School of Medicine and Sun Yat-sen University.

**Fig. 1:**
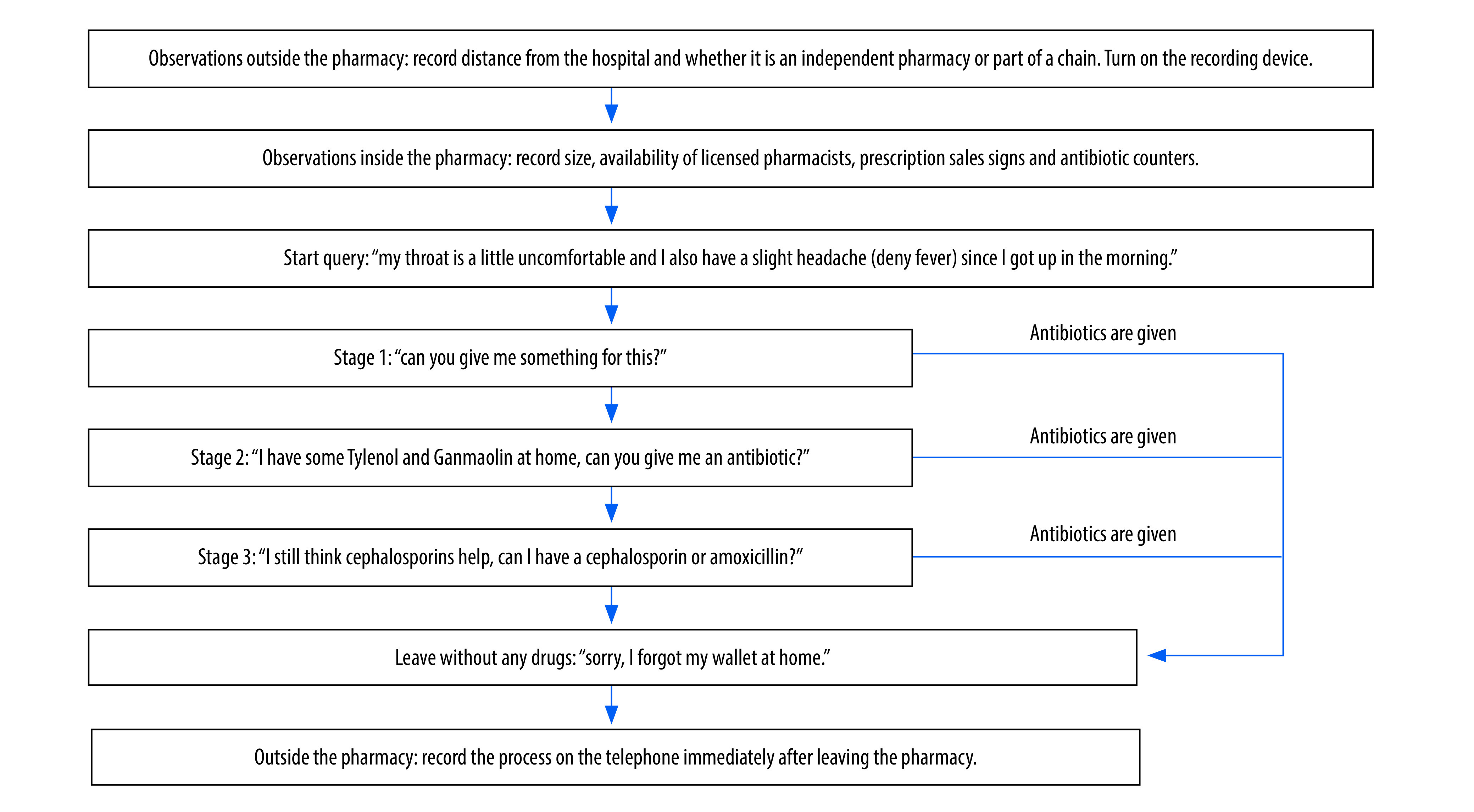
Flowchart of the steps taken by simulated clients, China, 2017 and 2021

Note: Tylenol® and Ganmaolin are recognized brand names for two commonly used over-the-counter medicines for fever and pain relief in China.

We conducted the surveys from July to September in 2017 and 2021. The medical students (simulated patients) went to the pharmacies selected and followed the three stages of the protocol (Fig. 1). We instructed students to describe the symptoms of a mild upper respiratory tract infection, to deny any symptoms of fever, not to display any visible symptoms and not to insist on being given antibiotics. When the pharmacy refused to sell antibiotics, the specific reason was recorded, including: a prescription was required; antibiotics were not indicated; no antibiotics were in stock; antibiotic sales were banned; or any other reasons.

### Analysis

We used descriptive statistics with frequencies and 95% confidence intervals (CIs). We used the *χ^2^* test to compare the characteristics of pharmacies. We used multivariable logistic regression analyses to compare the pharmacies that sold antibiotics without prescription with pharmacies that did not. We used R version 4.2.1 (R Foundation, Vienna, Austria) for all statistical analyses.

### Ethical approval

We obtained ethical approval for the study from the Institutional Ethical Clearance Committee, Zhejiang University School of Public Health (approval numbers ZGL201706–5 and ZGL202107–1). Because of the nature of the simulated patient method, the committee waived the need for consent of the pharmacies. The aim of the study was not to expose pharmacy violations, but to anonymously report the prevalence of the sale of antibiotics without a prescription.

## Results

The region, level and location characteristics of the pharmacies were similar in the two surveys (Table 1). In 2017, the simulated patients visited 1345 retail pharmacies, but complete data were available for only 1106. In 2021, among the 1204 pharmacies visited, 1090 had complete data. Most pharmacies had counters where antibiotics were dispensed – 83.4% (922/1106) in 2017 and 83.3% (908/1090) in 2021. Most pharmacies also had signs saying that prescriptions were required for antibiotics, which increased from 91.4% (1011/1106) in 2017 to 97.3% (1061/1090) in 2021 (*P*-value: < 0.001). In 2017, 43.9% (485/1106) of the pharmacies visited had a licensed pharmacist on duty, which increased to 65.5% (714/1090) in 2021 (*P*-value: < 0.001).

**Table 1:** Characteristics of the pharmacies visited, China, 2017 and 2021

Characteristic	No. (%)		*P* ^b^
	2017 (*n* = 1106)	2021 (*n* = 1090)	
**Region**			0.85
East	364 (32.9)	347 (31.8)	
Central	416 (37.6)	420 (38.5)	
West	326 (29.5)	323 (29.6)	
**Level**			0.42
City	367 (33.2)	354 (32.5)	
County	433 (39.2)	407 (37.3)	
Township or village	306 (27.7)	329 (30.2)	
**Near a hospital** ^a^			0.38
Yes	588 (53.2)	558 (51.2)	
No	518 (46.8)	532 (48.8)	
**Type of pharmacy**			0.15
Part of a chain	702 (63.5)	658 (60.4)	
Independent	404 (36.5)	432 (39.6)	
**Size**			< 0.001
Large (> 100 m^2^)	187 (16.9)	100 (9.2)	
Middle (50–100 m^2^)	636 (57.5)	605 (55.5)	
Small (< 50 m^2^)	283 (25.6)	385 (35.3)	
**Had a counter where antibiotics were dispensed**			1.00
Yes	922 (83.4)	908 (83.3)	
No	184 (16.6)	182 (16.7)	
**Had signs that antibiotics required a prescription**			< 0.001
Yes	1011 (91.4)	1061 (97.3)	
No	95 (8.6)	29 (2.7)	
**Had a pharmacist**			< 0.001
Licensed and on duty	485 (43.9)	714 (65.5)	
Licensed but not on duty	275 (24.9)	259 (23.8)	
Unlicensed	346 (31.3)	117 (10.7)	

^a^ Near a hospital means the pharmacy was within 2 km of a hospital.

^b^ *χ^2^* test.

**Fig. 2:**
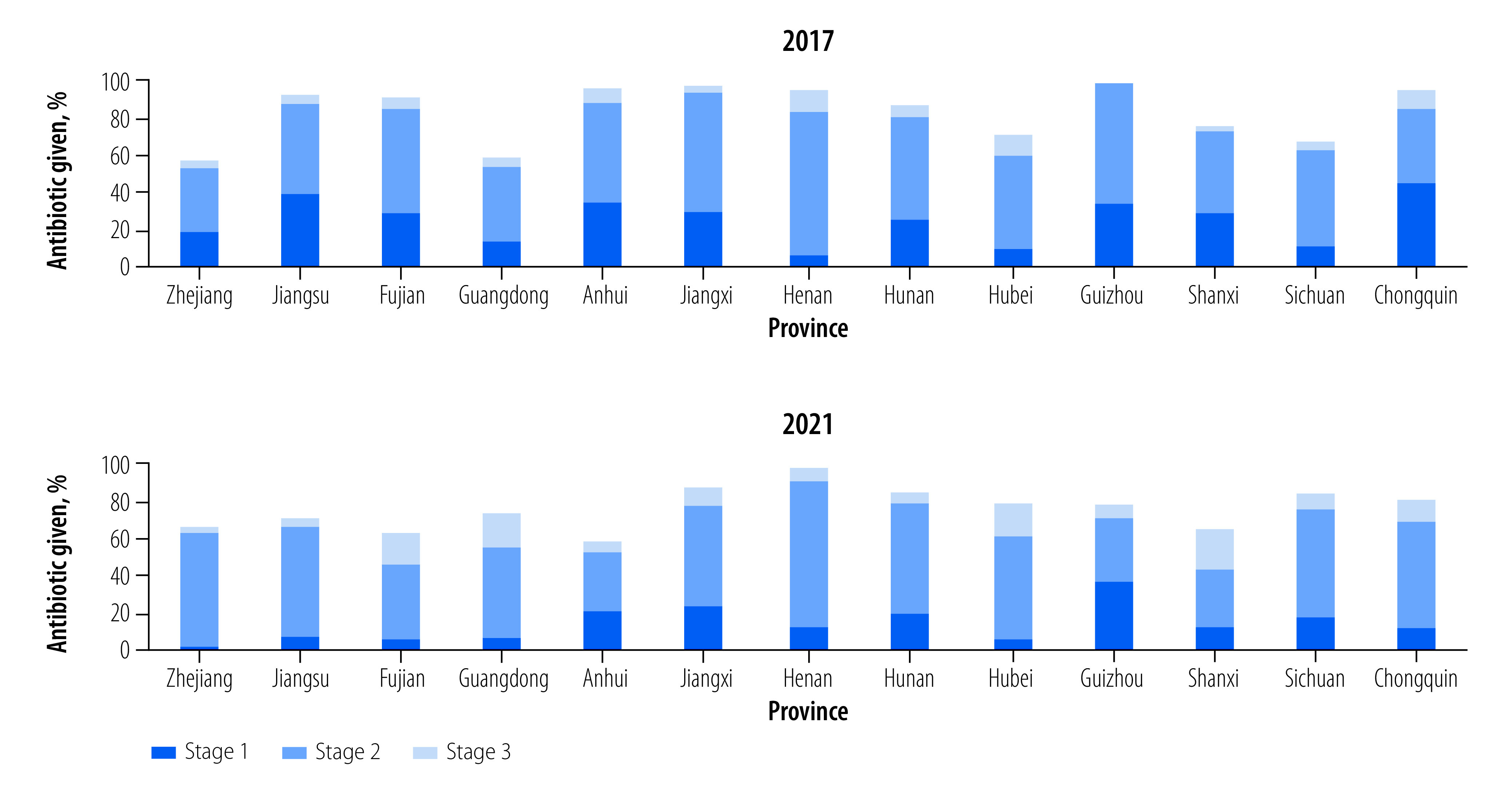
Sale of antibiotics without a prescription by stage and province , China, 2017 and 2021

Note: Stage 1: asked for medicine for a sore throat and headache; stage 2: asked for an antibiotic; stage 3: asked for amoxicillin or cephalosporin.

Sales of antibiotics without a prescription showed a small but significant decrease from 83.6% (925/1106; 95% CI: 81.5–85.8) in 2017 to 78.3% (853/1090; 95% CI: 75.9–80.8) in 2021 (*P*-value: 0.002; Table 2). Across the provinces, sales of antibiotics ranged from 57.0% (57/100) in Zhejiang to 98.8% (81/82) in Guizhou in 2017, and from 60.0% (48/80) in Anhui to 100% (85/85) in Henan in 2021 (Fig. 2 and Table 3 available at: https://www.who.int/publications/journals/bulletin/). The most common antibiotics sold were: penicillin (36.0%; 333/925 in 2017 and 49.4%; 421/853 in 2021); cephalosporins (29.6%; 274/925 in 2017 and 38.9%; 332/853 in 2021); and macrolides (27.0%; 250/925 in 2017 and 14.8%; 126/853 in 2021). After excluding pharmacies that were prohibited from selling antibiotics due to regional laws and regulations during COVID-19 (Shanxi, Anhui, Jiangsu, Zhejiang and Chongqing), sales of antibiotics without a prescription in 2017 and 2021 were not significantly different (83.6%; 925/1106; 95% CI: 81.5–85.8 in 2017 and 80.9%; 853/1054; 95% CI: 78.5–83.3 in 2021; *P*-value: 0.11; Table 2).

**Table 2:** Sales of antibiotics without a prescription at pharmacies, by stage, China, 2017 and 2021

Year	Total no.	Sales, no. (%)				*P*
		At stage 1	At stage 2	At stage 3	Overall	
2017	1106	279 (30.2)	576 (62.3)	70 (7.6)	925 (83.6)	–
2021	1090	157 (18.4)	571 (66.9)	125 (14.7)	853 (78.3)	0.002
2021^a^	1054	157 (18.4)	571 (66.9)	125 (14.7)	853 (80.9)	0.11

^a^ The results excluded cases where antibiotics could not be purchased because of coronavirus disease 2019.

Note: Stage 1: asked for medicine for a sore throat and headache; stage 2: asked for an antibiotic; stage 3: asked for amoxicillin or cephalosporin.

**Table 3:** Sales of antibiotics without a prescription at pharmacies, by stage and province, China, 2017 and 2021

Province	2017					2021			
	Overall (*n* = 1106)	At stage 1 (*n* = 279)	At stage 2 (*n* = 576)	At stage 3 (*n* = 65)		Overall (*n* = 1090)	At stage 1 (*n* = 157)	At stage 2 (*n* = 571)	At stage 3 (*n* = 121)
**East**									
Zhejiang	100	19	34	4		89	2	55	3
Jiangsu	92	36	45	4		81	6	49	4
Fujian	92	26	52	6		92	6	37	17
Guangdong	80	11	32	4		85	6	42	16
**Central**									
Anhui	92	32	49	7		80	17	26	5
Jiangxi	81	24	52	3		87	21	48	9
Henan	80	5	62	9		85	11	68	6
Hunan	83	21	46	5		84	17	51	5
Hubei	80	8	40	9		84	5	48	15
**West**									
Guizhou	82	28	53	0		79	30	27	6
Shanxi	83	24	37	2		84	11	27	18
Sichuan	81	9	42	4		79	15	46	7
Chongqing	80	36	32	8		81	10	47	10

Note: Stage 1: asked for medicine for a sore throat and headache; stage 2: asked for an antibiotic; stage 3: asked for amoxicillin or cephalosporin.

Most of the simulated patients were able to buy antibiotics when they asked directly for antibiotics at stage 2, 52.1% (576/1106) in 2017 and 52.4% (571/1090) in 2021 (*P*-value: 0.92). Others also obtained antibiotics at stage 1, 25.2% (279/1106) in 2017 and 14.4% (157/1090) in 2021 (*P*-value: < 0.001) and stage 3, 6.3% (70/1106) in 2017 and 11.5% (125/1090) in 2021 (*P*-value: < 0.001). The reasons for not offering antibiotics were: a prescription from a hospital was required (10.2%; 113/1106 in 2017 and 8.5%; 93/1090 in 2021); antibiotics were not indicated (5.2%; 58/1106 in 2017 and 8.9%; 97/1090 in 2021); no antibiotics were in stock (0.5%; 6/1106 in 2017 and 0.8%; 9/1090 in 2021); and antibiotic sales were banned during COVID-19 in the area (4.2%; 46/1090 in 2021). 

In both 2017 and 2021, pharmacies in central China were more likely to sell antibiotics without prescription than those in eastern China, OR: 2.61 (95% CI: 1.74–3.93) in 2017 and OR: 2.41 (95% CI: 1.68–3.47) in 2021 (Table 4). In 2017, pharmacies in western China were more likely to sell antibiotics without prescription than those in eastern China (OR: 1.74; 95% CI: 1.16 to 2.63), while no significant difference was seen in 2021 (Table 4). Regarding location, pharmacies in counties (OR: 1.96; 95% CI: 1.38–2.80) and at the township and village level (OR: 1.51; 95% CI: 1.03–2.20) were more likely to sell antibiotics than cities in 2021. In addition, pharmacies without antibiotics counters were less likely to sell antibiotics without a prescription in both 2017 and 2021, OR: 0.59 (95% CI: 0.37–0.94) in 2017 and OR: 0.66 (95% CI: 0.44–0.98) in 2021. Pharmacies without licensed pharmacists compared with pharmacies with a licenced pharmacist on duty were more likely to sell antibiotics in 2017, but not in 2021, OR: 2.00 (95% CI: 1.29–3.10) in 2017 and OR: 1.27 (95% CI: 0.76–2.10) in 2021. The association of distance from a hospital with sale of antibiotics without a prescription was weak in 2017, while no association was seen in 2021 (Table 4).

**Table 4:** Factors associated with sale of antibiotics without a prescription at pharmacies, China, 2017 and 2021

Variable	OR (95% CI)	
	2017	2021
**Region**		
East	Reference	Reference
Central	2.61 (1.74–3.93)	2.41 (1.68–3.47)
West	1.74 (1.16–2.63)	1.33 (0.93–1.91)
**Level**		
City	Reference	Reference
County	1.04 (0.71–1.54)	1.96 (1.38–2.80)
Township or village	1.61 (1.02–2.54)	1.51 (1.03–2.20)
**Near a hospital^a^**		
Yes	Reference	Reference
No	1.42 (1.01–2.00)	0.90 (0.66–1.22)
**Type of pharmacy**		
Part of a chain	Reference	Reference
Independent	1.07 (0.74–1.53)	0.75 (0.55–1.04)
**Size**		
Large (> 100 m^2^)	Reference	Reference
Middle (50–100 m^2^)	0.70 (0.43–1.15)	1.40 (0.85–2.29)
Small (< 50 m^2^)	0.79 (0.44–1.42)	1.16 (0.68–1.97)
**Had a counter where antibiotics were dispensed**		
Yes	Reference	Reference
No	0.59 (0.37–0.94)	0.66 (0.44–0.98)
**Had signs that antibiotics required a prescription**		
Yes	Reference	Reference
No	1.63 (0.79–3.39)	0.85 (0.34–2.12)
**Had a pharmacist**		
Licensed and on duty	Reference	Reference
Licensed but not on duty	1.02 (0.69–1.51)	1.11 (0.78–1.59)
Unlicensed	2.00 (1.29–3.10)	1.27 (0.76–2.10)

OR: odds ratio; CI: confidence interval; ref: reference category.

^a^ Near a hospital means the pharmacy was within 2 km of a hospital.

The two surveys in 2017 and 2021 showed similar quality of pharmacy services associated with antibiotics sales. Most pharmacies asked about the customer’s condition (65.4%; 723//1106) in 2017 and 66.1%; 721/1090 in 2021) and provided medication advice (71.0%; 785/1106 in 2017 and 70.7%; 771/1090 in 2021). However, few pharmacies asked the patient to provide a prescription (11.9%; 132/1106 in 2017 and 13.4%; 146/1090 in 2021), or asked the patient if they had seen a doctor (1.0%; 11/1106 in 2017 and 1.7%; 19/1090 in 2021; all *P*-values: > 0.05). But significantly more pharmacies in 2021 than in 2017 asked about drug allergies: 24.4% (270/1106) in 2017 and 30.9% (337/1090) in 2021 (*P*-value: < 0.001; Table 5).

**Table 5:** Quality of pharmacy services related to sale of antibiotics without a prescription, China, 2017 and 2021

Action	2017 (*n* = 1106)	2021 (*n* = 1090)	*P^a^*
**Asked about drug allergies**			< 0.001
Yes	270 (24.4)	337 (30.9)	
No	836 (75.6)	753 (69.1)	
**Asked about patient’s condition**			0.76
Yes	723 (65.4)	721 (66.1)	
No	382 (34.6)	369 (33.9)	
**Asked for a prescription**			0.33
Yes	132 (11.9)	146 (13.4)	
No	974 (88.1)	944 (86.6)	
**Provided medication advice**			0.94
Yes	785 (71.0)	771 (70.7)	
No	321 (29.0)	319 (29.3)	
**Asked if the patient had seen a doctor**			0.18
Yes	11 (1.0)	19 (1.7)	
No	1095 (99.0)	1071 (98.3)	

^a^ *χ^2^* test.

## Discussion

This study evaluated sales of antibiotics without a prescription in retail pharmacies in mainland China in 2017 and 2021, before and during COVID-19. In both surveys, healthy young adults with no actual symptoms were easily able to buy antibiotics without a prescription at a retail pharmacy. Overall, sales of antibiotics without a prescription slightly but significantly declined, regardless of the type, size, or other characteristics of the pharmacies. However, after excluding cases where antibiotics could not be purchased because of COVID-19, mainly in Shanxi, Anhui and Jiangsu, such sales did not decrease significantly between 2017 and 2021, even though stricter policies and regulations on antimicrobial resistance had been in place since 2020.^24^^,^^33^ In recent studies in China on sales of antibiotics without a prescription, these sales ranged from 70.1% (1690*/*2411) to 88.4% (130*/*147).^14^^,^^17^^,^^18^ Antibiotics without a prescription were found to be more easily obtained in western China (OR: 2.35; 95% CI: 1.27–4.34)^14^^,^^16^ and in rural areas (OR: 2.17; 95% CI: 1.32–3.57),^14^^,^^34^ which is in line with our results. Globally, studies on sales of antibiotics without a prescription have been done in many settings, especially in low- and middle-income countries.^8^^,^^35^ A systematic review and meta-analysis of studies in 24 countries where antibiotics were classed as prescription-only medicines, showed that the overall proportion of community pharmacies providing antibiotics without a prescription was 62% (95% CI: 53–72) which increased to 78% (95% CI: 59–97) if the patient requested them.^8^ A similar study in sub-Saharan African countries showed that 69% (95% CI: 58–80) of requests for antibiotics without a prescription in community pharmacies resulted in antibiotics being given.^35^

The sale of antibiotics may also have been influenced by COVID-19 regulations. Our survey in 2021 was conducted when COVID-19 had been brought under control after the first phase of the pandemic in China, when only small sporadic outbreaks were occurring. In 2021, most pharmacies visited asked clients to take their temperature and register individual information to assist with identification of suspected COVID-19 cases. In places with COVID-19 cases, strict lockdowns and movement restrictions were imposed. In some of these areas, people were able to leave their homes to buy medicines for upper respiratory tract symptoms (sometimes including antibiotics) in a pharmacy, thus avoiding the compulsory nucleic acid test in hospital.^36^^,^^37^ This situation may have increased the sale of antibiotics without a prescription in retail pharmacies. Such sales were mainly driven by profit,^38^ because of the need to make up for lost income during the pandemic.

Our two surveys identified some common issues. First, they illustrate the importance of the demand side in illegal antibiotic sales. Most simulated patients in our surveys were given antibiotics without a prescription when they asked directly for an antibiotic at stage 2. In 2021, the simulated patients had significantly less access to antibiotics at stage 1 and greater access at stage 3 compared with in 2017. Pharmacists would sell at the specific request of the patient. This finding may be partly explained by the results of a nationwide community survey in China, which showed that more than 60% of the public believed that antibiotics could treat viral diseases.^38^ National education campaigns and programmes are needed to raise public awareness of the risks of antibiotic misuse.

Second, the quantity and quality of pharmacists should be strengthened. Trained pharmacists are easily approachable professionals^39^ and can help patients choose suitable treatments.^14^^,^^40^^–^^42^ However, our study showed that more than half of pharmacies had no licensed pharmacist on duty in 2017, a number that decreased to about one third in 2021, even though by law a licensed pharmacist should be present for the sale of prescription drugs. In 2021, the total number of retail pharmacies in China was 586 530,^43^ but there was a shortage of licensed pharmacists. As for the quality of services, most of the pharmacists did not ask patients if they had seen a doctor, whether they had a prescription, or whether they had a history of drug allergy. About a third of the pharmacies did not enquire about the patient’s condition, nor did they offer any advice about other medication, with no changes from 2017 to 2021. To improve the quality of services, pharmacists need to be educated in basic prescribing practice. In addition, local health bureaus should take responsibility for monitoring pharmacists’ prescribing behaviour and improving pharmacy management.

A strength of our study was that the surveys were conducted with a standard procedure and on a large sample across China. Furthermore, we used the simulated patient method which is regarded as the gold standard for this type of research. Our study also had some limitations. First, we only included about 80 pharmacies per province. However, the pharmacies were purposively sampled to include a range of different categories of pharmacy. Second, we only conducted the study in 13 out of 31 provinces in mainland China and used just one specific disease description (mild upper respiratory tract infection); thus, generalization of the results should be made with caution.

Given the ease with which the public can obtain antibiotics without a prescription in pharmacies across China, despite the fact that legislation banning this practice has been in place for nearly 20 years, measures to strengthen good prescribing practice are needed. First, enforcement of the existing legislation is needed. This action could be the responsibility of local health bureaus, as noted above. Second, in parallel with this enforcement, a higher level of fines should be imposed for pharmacies that break the law. Evidence from other countries shows that fines can be an effective deterrent and result in compliance with legislation and regulations. For example, in Saudi Arabia, sales of antibiotics without a prescription decreased after fines were increased and regulations were enforced;^44^ however, in Viet Nam, increased fines did not significantly affect such sales.^45^ A system of standardized prescriptions could be developed with traceable authentication, for example, a quick response (QR) code. New technology such as tracking and tracing systems could also be applied.^46^^,^^47^ Third, a single regulation measure may not be sufficient, as a study in South Africa showed that a combination of regulations, including a ban on sales of antibiotics without a prescription, supervision of prescribing by licensed pharmacists, and sets of ethical and professional codes were necessary to change behaviour.^48^ Fourth, educational programmes for pharmacists, in parallel with education campaigns for the general public, need to be conducted. Finally, the authorities could also disclose the names of pharmacies selling antibiotics illegally, since social norms can help with behaviour modifications.^26^^,^^45^^,^^49^

To conclude, despite efforts by the government to tackle illegal sales of antibiotics in pharmacies, little has changed over the past 4 years. Public education campaigns and strong enforcement, combined with other measures, such as other legislation, are recommended to ensure antibiotics are only sold with a prescription in retail pharmacies in China.
